# Lipid-binding allergens from *Dermatophagoides pteronyssinus *mites extract isolated by liposomes

**DOI:** 10.1186/1753-6561-8-S4-P20

**Published:** 2014-10-01

**Authors:** Bianca Uliana Picolo, Ernesto Akio Taketomi, Jair Pereira Cunha-Junior

**Affiliations:** 1 Laboratory of Allergy and Clinical Immunology, Federal University of Uberlândia, Minas Gerais, Brazil

## Background

House dust mites (HDM), including *Dermatophagoides pteronyssinus *(Dpt) and *Dermatophagoides farinae *(Df), are one of the commonest aeroallergens worldwide eliciting allergic manifestations [1]. Allergenic phenomenon is tightly associated both with the mites themselves and with ligands derived from mite-associated bacterial/fungal products. Some *Dpt *allergens belong of lipid-binding proteins, including Der p 2 (protein with myeloid differentiation protein 2 (MD-2) related lipid recognition domain), Der p 7 (structurally homologous to lipid binding protein family) and Der p 13 (lipid transporter molecule) [2]. Thus, in this study we aimed to evaluate the ability of liposomes to adsorb lipid-binding proteins from *Dermatophagoites pteronyssinus *mites extract.

## Methods

Liposomes were prepared by ethanolic injection using dipalmitoyl phosphatidylcholine/cholesterol or dipalmitoyl phosphatidylethanolamine/ cholesterol diluted in ethanol. To Poll-down assays, Dpt allergens were incubate with liposome preparations and than washed three times with PBS solution. The adsorbed proteins on liposome surface were removed by SDS treatment and then analyzed by SDS-PAGE. Additionally, the lipid-binding proteins were analyzed by ELISA to evaluate the immunoreativity of Dpt*-*specific IgE and IgG1 antibodies [3].

## Results and conclusions

Several proteins ranging from 21 to 205 kDa were enriched in poll-down assays, including a polypeptide with high molecular weight (>205 kDa). In addition, Dpt-adsorbed on liposomes were reactive to IgE and IgG1 antibodies from allergic patients analyzed by ELISA. Further analysis using mass spectrometry will be conducted to identify the proteins adsorbed on liposome surface. Liposomes might be used to produce enriched fractions of lipid-binding proteins from *Dermatophagoides pteronyssinus *for further studies in allergic diseases.
